# Supplementary material for: The Expression Profiles of lncRNAs Are Associated with Neoadjuvant Chemotherapy Resistance in Locally Advanced, Luminal B-Type Breast Cancer
Source: Int J Mol Sci. 2024 Jul 24;25(15):8077. doi: 10.3390/ijms25158077 (PMC11311431; doi:10.3390/ijms25158077)
Supplement: Supplementary file 1 [file ijms-25-08077-s001.zip › ijms-3047584-supplementary.pdf]

International Journal of
Molecular Sciences

MDPI

Supplemental Figures

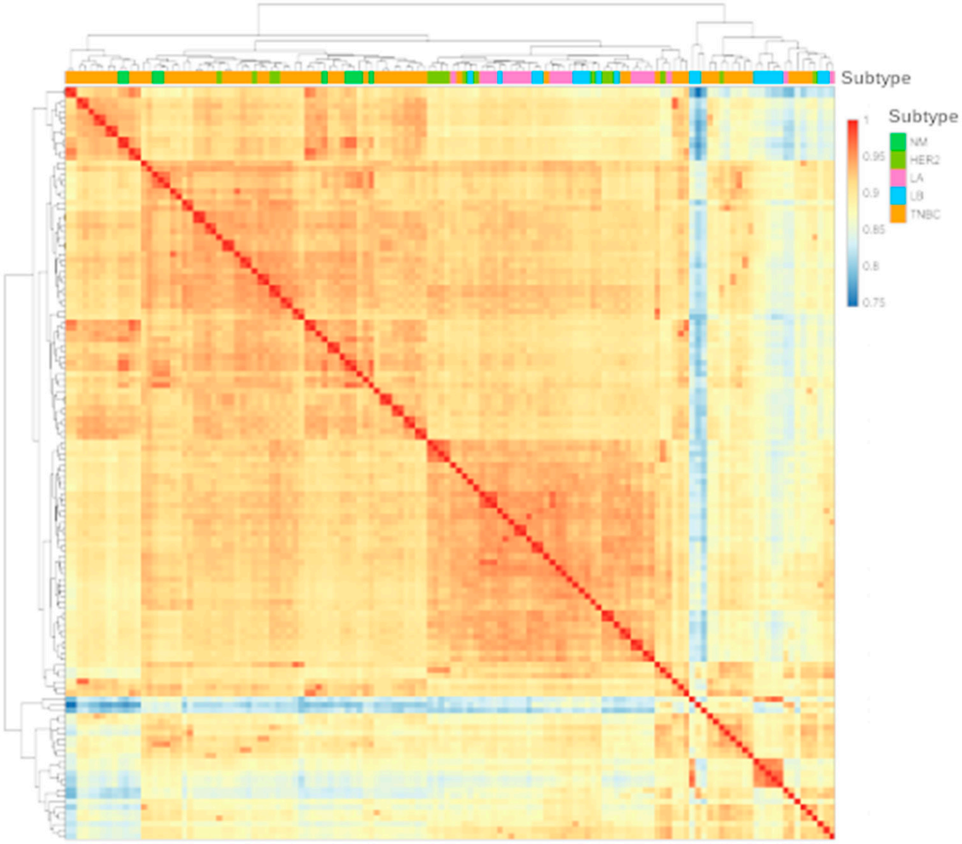

Supplemental Figure S1. Transcriptome analysis of 150 breast cancer cell lines from public databases. Heat map constructed from similarities matrix showing the clustering by transcriptome profile of 150 breast cancer cell lines according to their respective molecular subtype. NM: Normal; HER2: HER2 enriched cell lines; LA: Luminal A cell lines; LB: Luminal B cell lines; TNBC: Triple negative breast cancer cell lines.

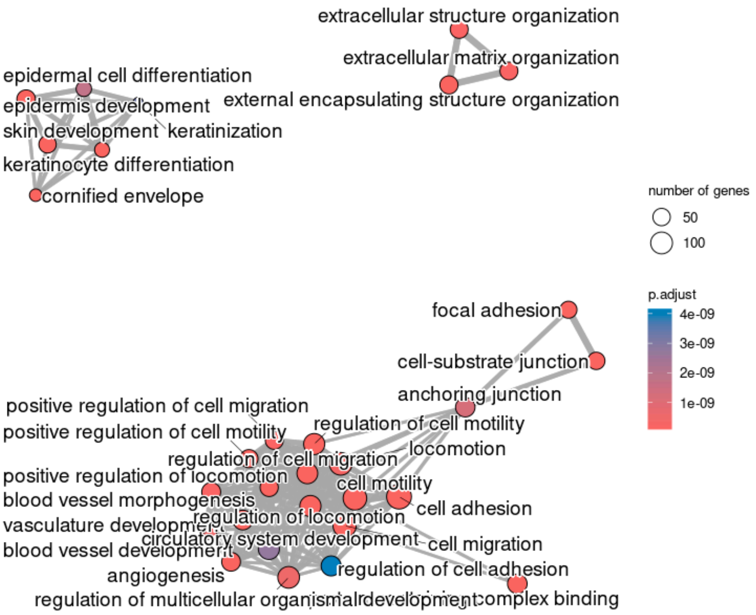

Supplemental Figure S2. The transcriptome profile of breast cancer cell lines is related to cell differentiation and motility. Enrichment map of the biological processes associated to the 2874 differentially expressed genes in breast cancer cell lines. The size of the dot is given by the number of genes matched for each gene set, and de dot color represent the adjusted p-value for the gene set enrichment.

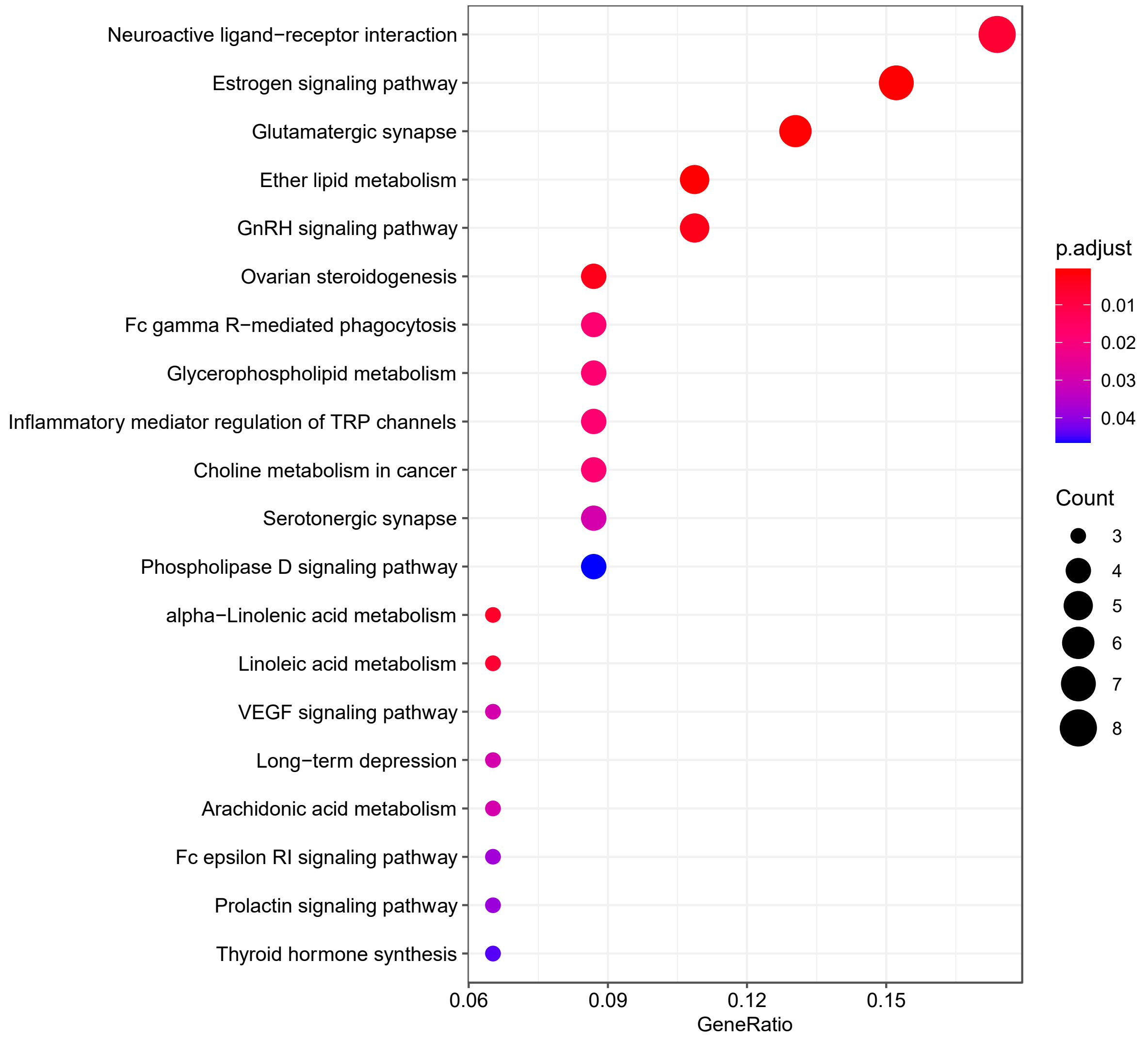

Supplemental Figure S3. NAC resistance-associated mRNAs and lncRNAs are related to neuron and hormone biological processes in luminal breast cancer tumors. Dot plot of the biological processes associated to differentially expressed lncRNAs in NAC resistant luminal tumors from GSE123845 and GSE163882 cohorts. The size of the dot is given by the number of genes matched for each gene set, and de dot color represent the adjusted p-value for the gene set enrichment.

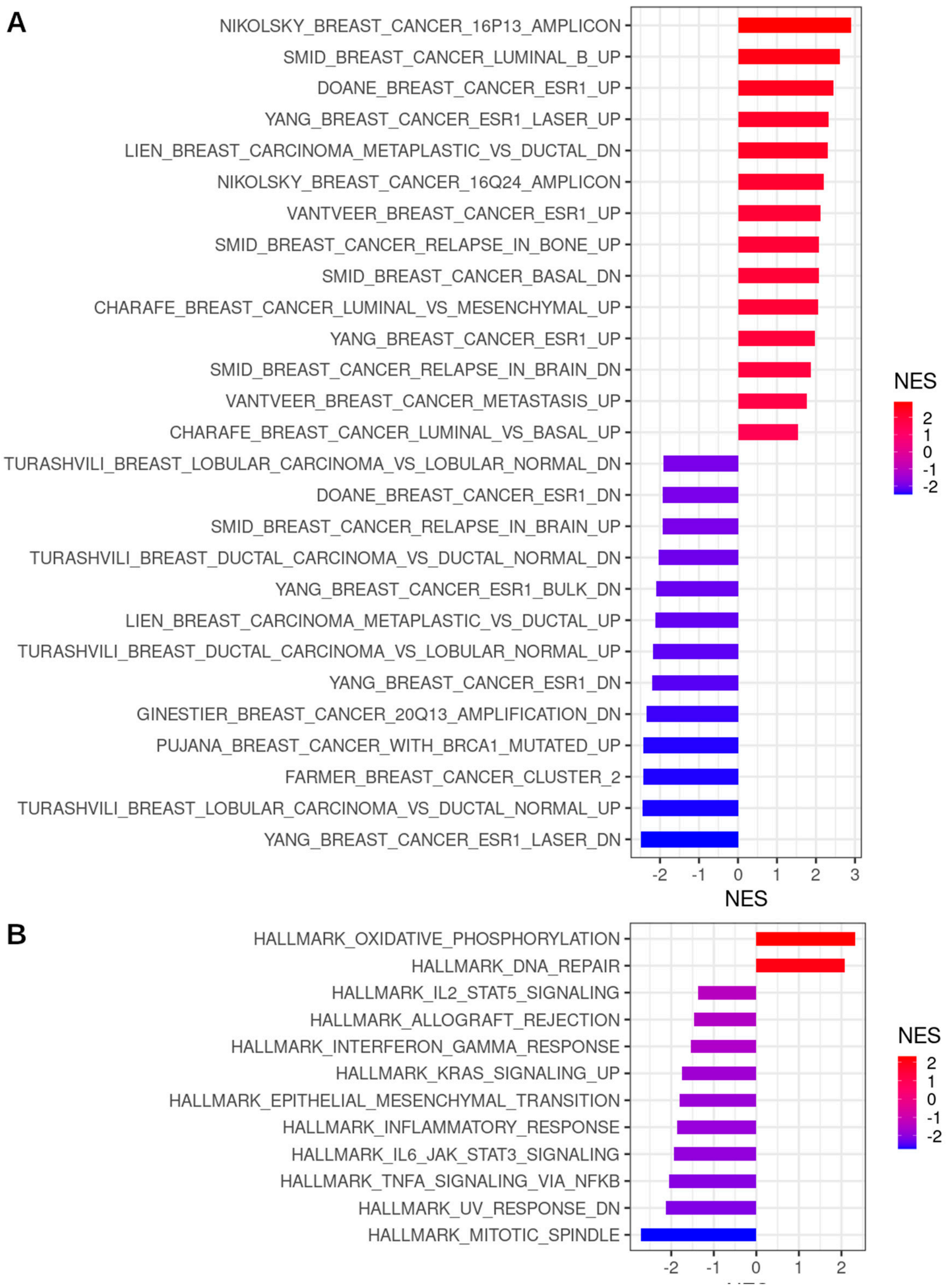

Supplemental Figure S4. NAC resistance-associated lncRNAs are related to neuron and hormone biological processes in luminal breast cancer tumors. A) Bar plot of the breast related gene sets associated to differentially expressed lncRNAs MAPT-IT1, GATA3-AS1, FAM222A-AS1 and FLJ12825 in NAC resistant luminal tumors from TCGA BRCA project cohort. B) Bar plot of the cancer hallmarks related gene sets associated to differentially expressed lncRNAs APT-IT1, GATA3-AS1, FAM222A-AS1 and FLJ12825 in NAC

resistant luminal tumors from TCGA BRCA project cohort. The color of the bar is given by the normalized enrichment score (NES). P value adjusted (FDR) < 0.05.

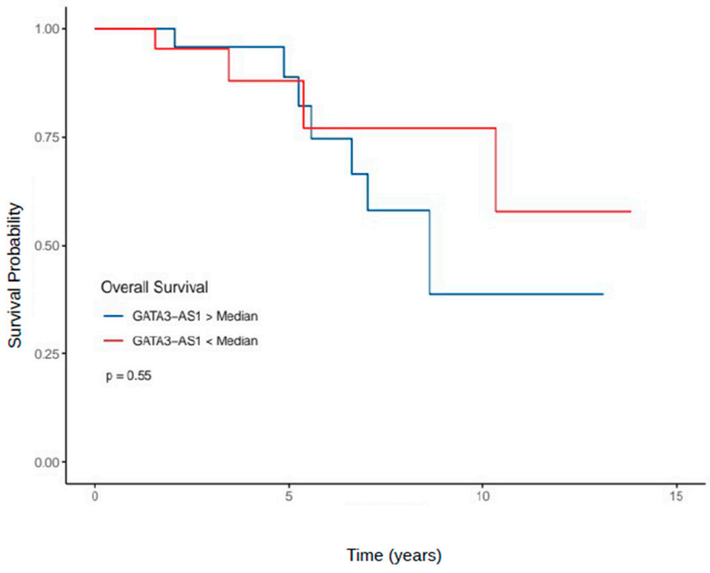

Supplemental Figure S5. The lncRNA GATA3-AS1 is not a prognostic biomarker for overall survival in luminal B LABC patients. Kaplan-Meier curve showing the overall survival for luminal B LABC patients after NAC treatment according to GATA3-AS1 expression (n = 49, GATA3-AS1 median = 0.027, p = 0.055).

Supplemental Tables

Supplemental Table S1: Top 40 lncRNAs differentially expressed in luminal B breast cancer patients.

**LncRNA Symbol**	**Log2 Fold Change**	**P value (adjusted)**
AL157387.1	23.74	3.20E-28
TUSC8	14.74	3.86E-15
TTC39A-AS1	13.71	6.56E-07
LINC02747	11.5	2.03E-16

LINC01488	9.95	1.19E-11
ST8SIA6-AS1	9.89	1.35E-13
AC093001.1	9.62	2.67E-13
AP005121.1	8.81	5.76E-06
LINC02303	8.54	7.77E-08
RMST	7.9	2.27E-06
AC055854.1	7.52	3.35E-09
AC069120.1	7.5	2.15E-10
CT62	7.15	1.18E-13
MIR9-3HG	6.75	3.33E-12
AL133370.1	6.22	6.64E-06
AC141930.1	6.12	1.25E-08
AC144450.1	6.02	1.20E-08
LINC02593	5.26	1.30E-09
AC010184.1	5.21	1.04E-10
AC110619.1	5.2	3.33E-09
HOXC-AS3	4.91	2.98E-07
AC006441.1	4.43	2.99E-05
DSCAM-AS1	4.37	8.56E-04
AC005288.1	3.61	2.83E-03
AC093838.1	3.59	5.01E-03
AC018865.2	3.34	4.33E-03
VIPR1-AS1	2.69	7.70E-04
AC127526.5	2.69	2.28E-03
GATA3-AS1	2.49	6.76E-03
MIR222HG	-5.22	6.51E-06

AC010198.2	-5.39	9.15E-08
HOXA-AS2	-5.84	4.07E-07
AC245041.2	-6.02	6.84E-06
AC006058.1	-6.07	2.07E-05
MYOSLID	-6.31	6.98E-13
LINC00707	-6.41	6.33E-10
AC093866.1	-6.89	1.76E-10
AL161431.1	-7.07	2.77E-09
HOTAIRM1	-7.69	5.65E-11
IGFL2-AS1	-7.96	3.72E-12

Table S2: Transcriptome breast cancer cell lines accession data.

**Cell line**	**SRR***
BT549	SRR12060750
CAL120	SRR12060751
CAL851	SRR12060753
CAMA1	SRR12060754
HCC1143	SRR12060755
HCC1395	SRR12060756
HCC1419	SRR12060757
HCC1428	SRR12060758
HCC1500	SRR12060759
HCC1806	SRR12060760
HCC1937	SRR12060761
HCC1954	SRR12060762
HME1	SRR12060765
MCF10A	SRR12060767
MCF7	SRR12060769
MDAMB134	SRR12060770
MDAMB361	SRR12060773
SKBR3	SRR12060783
SUM1315	SRR12060784
T47D	SRR12060787
BT474	SRR12060796
BT483	SRR12060798
HCC202	SRR12060814

ZR751(4)	SRR1313062
BT474	SRR1313063
HCC1599	SRR1313069
SKBR3(9)	SRR1313073
MDAMB134	SRR1313076
MDAMB361(1)	SRR1313078
ZR7530(1)	SRR1313080
HCC1569(1)	SRR1313088
SUM102	SRR1313089
AU565(6)	SRR2532318
BT474(1)	SRR2532320
BT483	SRR2532321
EFM192A	SRR2532330
EVSAT	SRR2532331
HCC202	SRR2532345
HCC2185	SRR2532346
HCC2218	SRR2532347
HCC712	SRR2532352
JIMT1	SRR2532355
MCF12A	SRR2532361
MDAMB175VII	SRR2532365
MDAMB330	SRR2532367
MDAMB415	SRR2532369
SUM1315	SRR2532380

*SRR codes were extracted from GEO Datasets.

For breast cancer tumor analysis, the transcriptome data cited in this study is listed below:

Korean breast cancer cohort: Park, Y.H.; Lal, S.; Lee, J.E.; Choi, Y.-L.; Wen, J.; Ram, S.; Ding, Y.; Lee, S.-H.; Powell, E.; Lee, S.K.; et al. Chemotherapy Induces Dynamic Immune Responses in Breast Cancers That Impact Treatment Outcome. Nat Commun 2020, 11, 6175, doi:10.1038/s41467-020-19933-0.

US breast cancer cohort: Chen, J.; Hao, L.; Qian, X.; Lin, L.; Pan, Y.; Han, X. Machine Learning Models Based on Immunological Genes to Predict the Response to Neoadjuvant Therapy in Breast Cancer Patients. Front Immunol 2022, 13, 948601, doi:10.3389/fimmu.2022.948601.

TCGA Breast Cancer cohort: Weinstein, J.N.; Collisson, E.A.; Mills, G.B.; Shaw, K.R.M.; Ozenberger, B.A.; Ellrott, K.; Shmulevich, I.; Sander, C.; Stuart, J.M. The Cancer Genome Atlas Pan-Cancer Analysis Project. Nat Genet 2013, 45, 1113–1120, doi:10.1038/ng.2764.

SCAN-B cohort: Saal, L.H.; Vallon-Christersson, J.; Häkkinen, J.; Hegardt, C.; Grabau, D.; Winter, C.; Brueffer, C.; Tang, M.-H.E.; Reuterswärd, C.; Schulz, R.; et al. The Sweden Cancerome Analysis Network - Breast (SCAN-B) Initiative: A Large-Scale Multicenter Infrastructure towards Implementation of Breast Cancer Genomic Analyses in the Clinical Routine. Genome Med 2015, 7, 20, doi:10.1186/s13073-015-0131-9.

For Hispanic breast cancer tumor analysis, the transcriptome data cited is in GEO datasets: GSE270967.
